# PEP-1-PON1 Protein Regulates Inflammatory Response in Raw 264.7 Macrophages and Ameliorates Inflammation in a TPA-Induced Animal Model

**DOI:** 10.1371/journal.pone.0086034

**Published:** 2014-01-23

**Authors:** Mi Jin Kim, Hoon Jae Jeong, Dae Won Kim, Eun Jeong Sohn, Hyo Sang Jo, Duk-Soo Kim, Hyun Ah Kim, Eun Young Park, Jong Hoon Park, Ora Son, Kyu Hyung Han, Jinseu Park, Won Sik Eum, Soo Young Choi

**Affiliations:** 1 Department of Biomedical Science and Research Institute of Bioscience and Biotechnology, Hallym University, Chunchon, Gangwondo, Korea; 2 Department of Biochemistry and Molecular Biology, Research Institute of Oral Sciences, College of Dentistry, Kangnung-Wonju National University, Gangneung, Gangwondo, Korea; 3 Department of Anatomy, College of Medicine, Soonchunhyang University, Cheonan-Si, Chungcheonnamdo, Korea; 4 Division of Rheumatology, Department of Internal Medicine, Hallym University Sacred Heart Hospital, Pyongchon, Kyunggido, Korea; 5 Department of Biological Sciences, Sookmyung Women’s University, Seoul, Korea; National Institutes of Health, United States of America

## Abstract

Paraoxonase 1 (PON1) is an antioxidant enzyme which plays a central role in various diseases. However, the mechanism and function of PON1 protein in inflammation are poorly understood. Since PON1 protein alone cannot be delivered into cells, we generated a cell permeable PEP-1-PON1 protein using protein transduction domains, and examined whether it can protect against cell death in lipopolysaccharide (LPS) or hydrogen peroxide (H_2_O_2_)-treated Raw 264.7 cells as well as mice with 12-O-tetradecanoyl phorbol-13-acetate (TPA)-induced skin inflammation. We demonstrated that PEP-1-PON1 protein transduced into Raw 264.7 cells and markedly protected against LPS or H_2_O_2_-induced cell death by inhibiting cellular reactive oxygen species (ROS) levels, the inflammatory mediator’s expression, activation of mitogen-activated protein kinases (MAPKs) and cellular apoptosis. Furthermore, topically applied PEP-1-PON1 protein ameliorates TPA-treated mice skin inflammation via a reduction of inflammatory response. Our results indicate that PEP-1-PON1 protein plays a key role in inflammation and oxidative stress *in vitro* and *in vivo*. Therefore, we suggest that PEP-1-PON1 protein may provide a potential protein therapy against oxidative stress and inflammation.

## Introduction

Paraoxonase 1 (PON1) is a member of the *PON* gene family (PON1, PON2 and PON3), which are located on chromosome 7(q21.22) and are mainly synthesized in the liver and widely distributed in tissues including the liver, kidney, and intestine. PON1 is a calcium-dependent esterase that is known to hydrolyze organophosphates and pesticides [1]–[6]. PON1 is associated with high-density lipoprotein (HDL) and inhibits low-density lipoprotein (LDL) oxidation. Thus, it is considered as an antioxidant enzyme [6]–[8]. Several studies have shown that PON1 knockout mice are susceptible to the atherosclerosis, whereas overexpression of PON1 in mice reduced atherosclerosis [9]–[11]. In addition, the association between PON1 and various human diseases including heart disease, Parkinson’s disease, and diabetes is well documented [11]–[14].

Lipopolysaccharide (LPS) is a well known gram-negative bacterial outer membrane component, which triggers the inflammatory response and production of pro-inflammatory mediators such as cyclooxygenase-2 (COX-2), cytokines (interleukin-1 beta; IL-1β and IL-6), tumor necrosis factor-alpha (TNF-α) and reactive oxygen species (ROS). These inflammatory mediators are closely associated with the pathogenesis of various inflammatory diseases [15]–[18]. Also, generated ROS alter the structure and function of cells and contributes to cell death [19]–[20].

The use of protein as therapeutic agents is limited by their molecular size, low permeability and biochemical characteristics [21], [22]. However, many studies have shown that the delivery of therapeutic proteins to cells and tissues using protein transduction domains (PTDs) is a powerful tool in clinical protein application [21]–[27]. In previous studies, we showed that PTD fusion proteins transduced into cells and tissues as well as protected against various diseases including skin inflammation and neuronal diseases [28]–[36].

In the present study, we investigated whether PEP-1-PON1 transduced into cells and tissues as well as whether or not it protected against LPS or H_2_O_2_-induced inflammation and oxidative stress. Our results show that PEP-1-PON1 efficiently transduced into Raw 264.7 cells and markedly protected against LPS- or H_2_O_2_-induced inflammation and cell death. Furthermore, topically applied PEP-1-PON1 led to a significant improvement in 12-O-tetradecanoylphorbol-13-acetate (TPA)-induced ear edema suggesting that PEP-1-PON1 could be a potential therapeutic agent for various inflammation and oxidative stress-related diseases.

## Materials and Methods

### Ethics Statement

All experimental procedures involving animals and their care conformed to the Guide for the Care and Use of Laboratory Animals of the National Veterinary Research and Quarantine Service of Korea and were approved by the Hallym Medical Center Institutional Animal Care and Use Committee.

### Materials

FBS and antibiotics were purchased from Gibco BRL (Grand Island, NY, USA). LPS and TPA were purchased from Sigma-Aldrich (St. Louis, MO, USA). Synthetic PEP-1 peptides used in this experiment were acquired from PEPTRON (Daejeon, Korea). Human PON1 cDNA was isolated using the polymerase chain reaction (PCR) technique. Cell Signaling Technology (Beverly, MA, USA) and Santa Cruz Biotechnology (Santa Cruz, CA, USA) provided the indicated primary antibodies. PCR primers were purchased from Bioneer (Seoul, Korea). Remaining chemicals and reagents were of the best possible commercial grade.

### Construction of PEP-1-PON1 Plasmid

In a previous study, we constructed a PEP-1 expression vector [28]. To construct a cell permeable PEP-1-PON1 protein, polymerase chain reaction (PCR) was used to amplify the cDNA sequence of human PON1 (GenBank: BC074719.2) using the following primers; sense primer 5′-CTCGAGGCGAAGCTGATTGCG-3′; antisense primer, 5′-GGATCCTTAGAGCTCACAGTAAAGAGC-3′. The PCR product was subcloned into a TA cloning vector and ligated into the PEP-1 expression vector, resulting in PEP-1-PON1 expression vector. A PON1 expression vector was also constructed without the PEP-1 to be used as a control.

### Expression and Purification of PEP-1-PON1 Proteins

After the plasmids were transformed into *E. coli* BL21 (DE3) cells, the PEP-1-PON1 and control PON1 proteins were induced by adding 0.5 mM isopropyl-β-D-thio-galactoside (Duchefa, Haarlem, Netherlands) at 37°C for 6 h. Recombinant proteins obtained from harvested cells were lysed by sonication after which the proteins were purified by Ni^2+^-nitrilotriacetic acid Sepharose affinity column chromatography (Qiagen, Valencia, CA, USA) and PD-10 column chromatography (Amersham, Brauncschweig, Germany) according to the manufacturer’s instructions [28]–[36]. To remove endotoxins of proteins, purified control PON1 and PEP-1-PON1 were treated with a Detoxi-Gel™ endotoxin removing gel (Pierce, Rockford, IL, USA) as per manufacturer’s instructions [34]. The Bradford assay was used to estimate protein concentration [37].

### Cell Culture and Transduction of PEP-1-PON1 Protein

Murine macrophage cell line Raw 264.7 cells were obtained from the Korean Cell Line Research Foundation (Seoul, Korea). The cells were cultured at 37°C under humidified conditions of 95% air and 5% CO_2_ in Dulbecco’s modified Eagle’s medium (DMEM) containing 20 mM HEPES/NaOH (pH 7.4), 5 mM NaHCO_3_, 10% fetal bovine serum (FBS) and antibiotics (100 µg/ml streptomycin, 100 U/ml penicillin).

To examine the transduction ability of PEP-1-PON1 and control PON1 protein, cells were grown on a 60 mm dish for 12 h. Then various concentrations (0.1–0.3 µM) of the proteins were added to the culture medium for 1 h. Also, the proteins (0.3 µM) were treated at various times (10–60 min) after which cells were treated with trypsin-EDTA and washed with phosphate-buffered saline (PBS) and harvested for the preparation of cell extracts.

For immunofluorescence analysis, cells were grown on coverslips and treated with PEP-1-PON1 protein (0.3 µM) for 1 h at 37°C. The cells were washed and fixed with 4% paraformaldehyde. The primary antibody (His-probe, Santa Cruz Biotechnology) was diluted 1∶2000, and incubated for 2 h at room temperature. The secondary antibody (Alexa fluor 488, Invitrogen) was diluted 1∶15000, and incubated for 1 h at room temperature in the dark. Nuclei were stained for 2 min with 1 µg/ml DAPI (Roche, Basel, Switzerland). The cellular localization of transduced PEP-1-PON1 protein was analyzed by confocal microscopy using a FV-300 microscope (Olympus, Tokyo, Japan) [33].

### RNA Isolation and Reverse Transcription (RT)-PCR Analysis

Total RNA of Raw 264.7 cells and ear biopsies was isolated using an Easy blue kit according to the manufacturer’s instructions (Invitrogen, Carlsbad, CA, USA). The RNA (2 µg) was then reversibly transcribed, and cDNA aliquots were amplified with the following specific primers. COX-2 antisense, 5′-TGGACGAGGTTTTTCCACC AG-3′; COX-2 sense, 5′-CAAAGGCCTCCATTGACCAGA-3′; TNF-α antisense, 5′-TGGCACCACTAGTTGGTTGTCTTT-3′; TNF-α sense, 5′-AAGTTCCCAAATGGCC TCCC-3′; IL-1β antisense, 5′-GTGCTGCCTAATGTCCCCTTGAATC-3′; IL-1β sense, 5′-TGCAGAGTTCCCCAACTGGTACATC-3′; IL-6 antisense, 5′-TGGATGGTCTTG GTCCTTAGCC-3′; IL-6 sense, 5′-CAAGAAAGACAAAGCCAGAGTCCTT-3′; β-actin antisense, 5′-GGACAGTGAGGCCAGGATGG-3′; β-actin sense, 5′-AGTGTG ACGTTGACATCCGTAAAGA-3′; and GAPDH antisense, 5′-AGTGATGGCATGGA CTGTGGTCAT-3′; GAPDH sense, 5′-ACCCCTTCATTGACCTCAACTACA-3′. A PCR Premix kit (Intron Biotechnology, Seoul, Korea) was used to perform PCR as per the manufacturer’s instructions. PCR products were resolved on a 1% agarose gel, after ethidium bromide staining, and were visualized with ultraviolet light [31]–[33].

### Assay for Cell Viability

To examine the protective effects of PEP-1-PON1 on Raw 264.7 cells exposed to H_2_O_2_, we performed a 3-(4,5-dimethylthiazol-2-yl)-2,5-diphenyltetrazolium bromide (MTT) assay as previously described [31]–[33]. Raw 264.7 cells were seeded into 96-well culture plates at 70% per well and were grown for 12 h. After the medium was replaced, cells were pretreated with PEP-1-PON1 protein (0.1–0.3 µM), control PON1 protein (0.3 µM), or PEP-1 peptide (0.3 µM) 1 h prior to treatment with H_2_O_2_ (1 mM and 1.5 mM). After incubation of cells for 16 h, cell viability was assessed using MTT. The absorbance was measured using an ELISA plate reader (Labsystems Multiskan MCC/340) at 570 nm and cell viability was defined as the % of control cells.

### Measurement of Intracellular ROS Levels

Intracellular ROS levels were measured using the ROS-sensitive fluorescent dye 2′,7′-dichlorofluorescein diacetate (DCF-DA) as described previously [29]–[31], [33]. After Raw 264.7 cells were incubated with PEP-1-PON1 protein (0.3 µM) for 1 h, they were treated with different concentrations of LPS (10 ng/ml for 50 min or 1 µg/ml for 30 min). Also, the cells were treated with different concentrations of H_2_O_2_ (1 mM for 20 min or 2 mM for 10 min). After having been washed twice with PBS, the cells were incubated with DCF-DA (20 µM) for 30 min at 37°C. Then intracellular fluorescence was measured at 485 nm excitation and 538 nm emission using a Fluoroskan ELISA plate reader (Labsystems Oy, Helsinki, Finland). Also, intracellular fluorescence images were taken using a fluorescence microscope (Nikon eclipse 80i, Japan).

### Measurement of DNA Protection

Terminal deoxynucleotidyl transferase (TdT)-mediated biotinylated UTP nick end labeling (TUNEL) staining was performed as described previously [29], [33]. Briefly, the cells were incubated with PEP-1-PON1 (0.3 µM) for 1 h, and then treated with H_2_O_2_ (1 mM for 15 h or 5 mM for 4 h). A Cell Death Detection kit (Roche Applied Science) was used to perform TUNEL staining as described in the manufacturer’s instructions. An Eclipse 80i fluorescence microscope (Nikon, Tokyo, Japan) was used to take images.

### Measurement of Mitochondrial Membrane Potential (MMP)

MMP staining was performed using a 5,5′,6,6′-tetrachloro-1,1′,3,3′-tetraethyl benzimidazolyl-carbocyanine iodide (JC-1) kit (Cayman, MI, USA) according to the manufacturer’s instructions [29], [33]. JC-1 is a cationic fluorescent dye that accumulates in mitochondria in a potential dependent manner. When MMP is high, JC-1 accumulates in the mitochondria and forms aggregates demonstrating red fluorescence. When MMP is low, JC-1 exists in the cytoplasm as monomers displaying green fluorescence. Briefly, the cells were incubated with PEP-1-PON1 protein (0.3 µM) for 1 h, and then exposed to H_2_O_2_ (2 mM) for 30 min. As above, images were produced using a fluorescence microscope (Nikon eclipse, 80i, Japan).

### Experimental Animal and Skin Inflammation

Male ICR mice (6–8 weeks, 22–26 g) were obtained from the Hallym University Experimental Animal Center. ICR mice were housed at 23°C and a relative humidity of 60%. They were exposed to a 12 h light: 12 h dark cycle and had free access to food and water.

12-O-tetradecanoylphorbol-13-acetate (TPA)-induced skin inflammation was carried out as described previously [33], [35]. Mice (n = 5, each group) were divided into the following five groups: control, TPA-treated, TPA+PON1-treated, TPA+PEP-1-PON1-treated, and TPA+PEP-1 peptide-treated. Briefly, inflammation was induced by using TPA (1.0 µg) dissolved in 20 µl of acetone applied to the surface of mouse ears every day for 3 days. PEP-1-PON1 protein and PON1 protein (10 µg/20 µl) were applied to the mouse ears 1 h after TPA treatment. As control, the same quantity of acetone (20 µl) was applied to mouse ears. After the last treatment, ear thickness was measured using a digital thickness gauge (Mitutoyo Corporation, Toyko, Japan). After mice were sacrificed, a punch (Kai Industries, Gifu, Japan) was used to obtain 5 mm ear biopsies from each animal. The biopsies were then weighed. To perform histological analysis, the biopsies were fixed in 4% paraformaldehyde and embedded in paraffin. The samples were then sectioned at a 5 µm thickness and stained with hematoxylin and eosin [33], [35].

### Western Blot Analysis

Western blot analysis was performed as described previously [38]. Sample proteins were prepared from Raw 264.7 cells by incubating cells in lysis buffer at 4°C for 1 h. Ear biopsies were homogenized vigorously in tissue protein extraction buffer with a protease inhibitor cocktail. Equal amounts of sample proteins were loaded onto a 12% sodium dodecyl sulfate-polyacrylamide gel electrophoresis (SDS-PAGE) and transferred to a nitrocellulose membrane, which was blocked with 5% nonfat dry milk in a TBS buffer containing 0.1% Tween 20 for 1 h. The membrane was probed with the indicated antibodies and the immunoreactive bands were visualized by ECL according to the manufacturer’s instructions. The band intensity was quantitated by densitometry using Image J software (NIH, Bethesda, MD, USA).

### Statistical Analysis

Data represent the mean of three experiments ± SD. Significant differences in means were assessed using student’s *t*-test and one-way ANOVA between the groups. Differences were considered to be significant at P<0.05.

## Results

### Purification and Transduction of PEP-1-PON1 Protein into Raw 264.7 Cells

**Figure 1:**
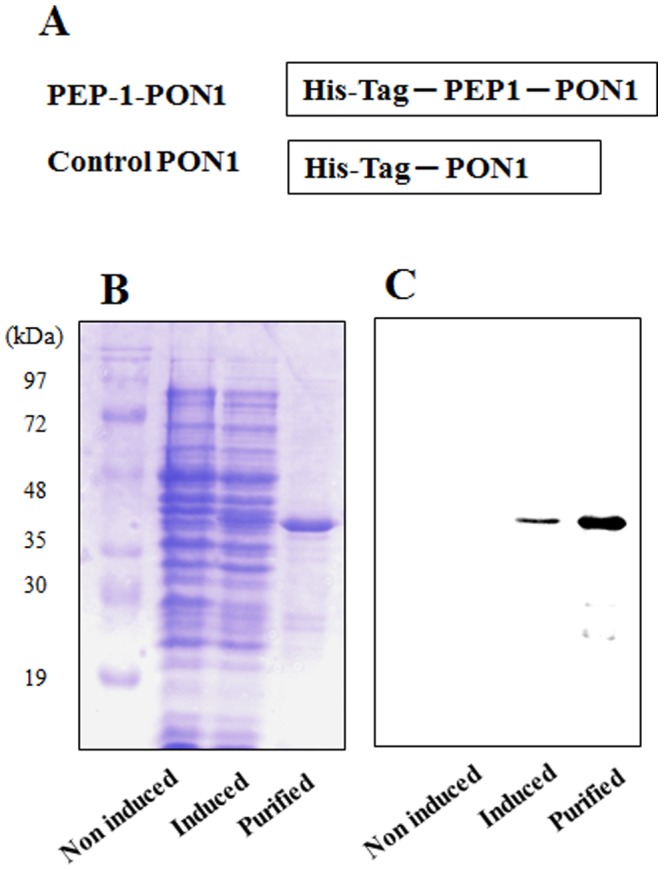
Construction and purification of PEP-1-PON1 protein. A representative diagram of the constructed PEP-1-PON1 protein (A). PEP-1-PON1 protein contains a His tag consisting of six histidine residues. Expressed and purified fusion proteins were analyzed by 12% SDS-PAGE (B) and subjected to Western blot analysis with an anti-rabbit polyhistidine antibody (C).

As shown in Fig. 1A, PEP-1-PON1 protein was produced by fusing a human PON1 gene with PEP-1 peptide a protein transduction domain. PEP-1-PON1 proteins were expressed in *E. coli* adding IPTG and purified using Ni^2+^-affinity chromatography. Additionally, PEP-1-PON1 proteins were further purified using Detoxi-Gel™ endotoxin removing gel. The expressed and purified proteins were then confirmed by SDS-PAGE and Western blot analysis using an anti-rabbit polyhistidine antibody (Figs. 1B and 1C).

**Figure 2:**
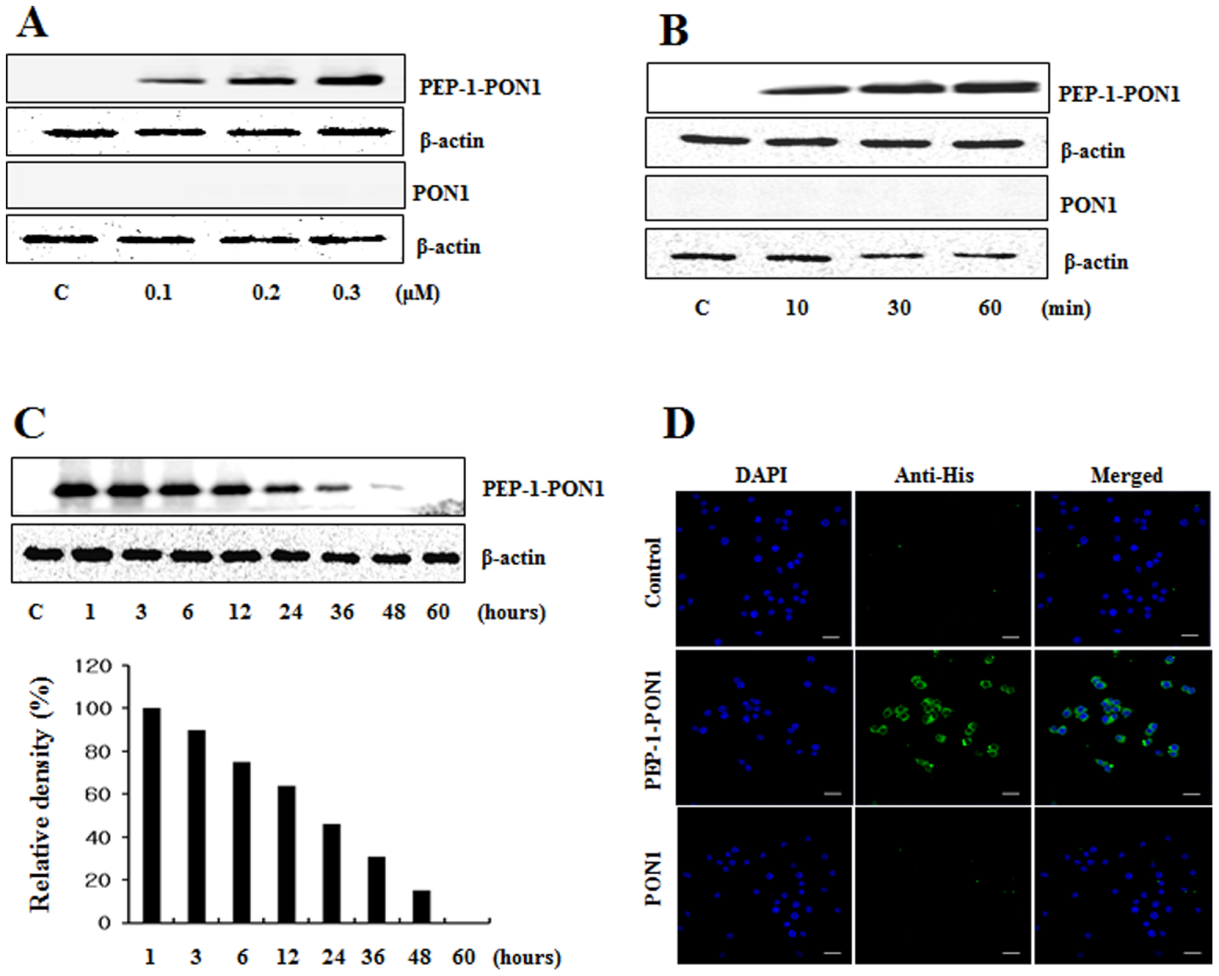
Transduction of PEP-1-PON1 proteins into Raw 264.7 cells. Cells were cultured in a 60-1-PON1 (0.1–0.3 µM) and control PON1 proteins were added to the culture media for 1 h (A), PEP-1-PON1 (0.3 µM) and control PON1 proteins were added to the culture media for 10–60 min (B), and analyzed by Western blot analysis. Transduction of PEP-1-PON1 stability was assessed after various time periods (1–60 h). Cells pretreated with 0.3 µM PEP-1-PON1 for 1 h and analyzed by Western blot analysis (C). The intracellular distribution of the transduced PEP-1-PON1 was observed by confocal microscopy (D). Scale bar = 20 µm.

To determine whether PEP-1-PON1 proteins are able to transduce into Raw 264.7 cells, cells were exposed to various concentrations (0.1–0.3 µM, 1 h) of PEP-1-PON1 proteins and over various times (10–60 min, 0.3 µM). Then, transduction levels were analyzed by Western blotting with an anti-His antibody. As shown in Fig. 2A and 2B, Western blot analysis revealed that PEP-1-PON1 proteins transduced into the cells in a dose- and time-dependent manner whereas, control PON1 protein did not transduce into the cells. Since stability is an important factor in protein therapy, we examined the stability of transduced PEP-1-PON1 proteins. Cells were treated with PEP-1-PON1 proteins at various time periods and the transduction levels were analyzed by Western blotting. Fig. 2C, shows that a significant level of transduced PEP-1-PON1 proteins persisted in the cells for 36 h. In addition, the cellular localization of transduced PEP-1-PON1 proteins in the cells was determined by immunofluorescence staining. The fluorescence signal was increased in PEP-1-PON1 protein treated cells. However, control PON1 treated cells were similar to normal control cells (Fig. 2D). The results of this experiment demonstrate that PEP-1-PON1 proteins efficiently transduced into cells and persisted for 36 h after transduction.

### Transduced PEP-1-PON1 Proteins Inhibits on LPS-induced Inflammatory Response

**Figure 3:**
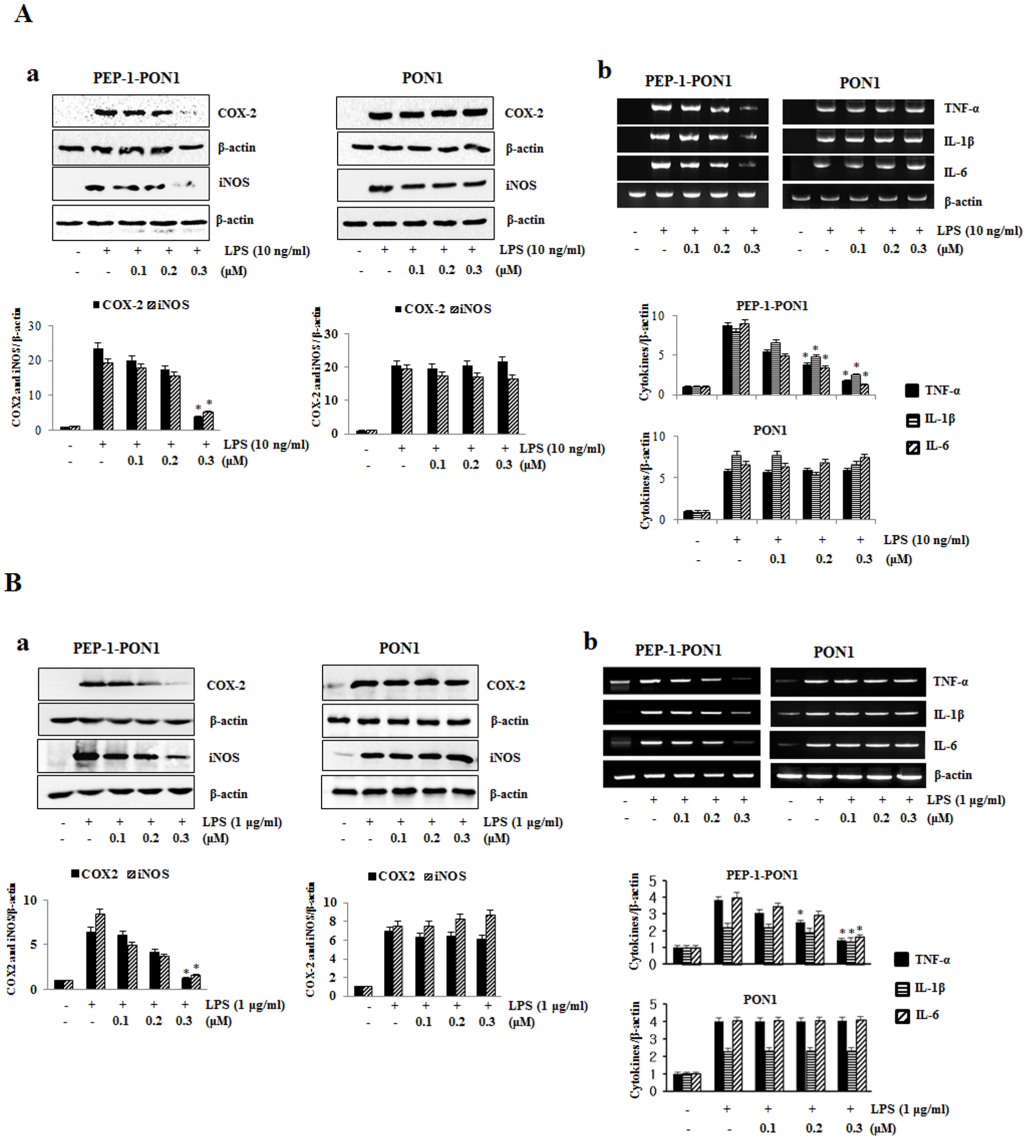
Inhibitory effect of PEP-1-PON1 protein on LPS-induced inflammatory response in Raw 264.7 cells. Raw 264.7 cells were stimulated with LPS with or without pretreatment with PEP-1-PON1 protein for 1 h. Cell lysates (A: 10 ng/ml LPS for 24 h, B: 1 µg/ml LPS for 12 h) were prepared and analyzed for COX-2 and iNOS protein expression by Western blotting and band intensity by densitometer (a). After total RNA was extracted from the cells (10 ng/ml and 1 µg/ml LPS for 24 h), cytokines (IL-1β, L-6, TNF-α) and β-actin mRNA were analyzed by RT-PCR using specific primers and band intensity by densitometer (b). *P<0.01, compared with LPS treated cells.

To examine the protective effects of PEP-1-PON1 protein on LPS-induced inflammation response, we assessed COX-2, iNOS, and cytokine (TNF-α, IL-1β, and IL-6) expression levels. After Raw 264.7 cells were treated with PEP-1-PON1 protein for 1 h, the cells were incubated for 24 h with 10 ng/ml LPS. LPS-induced COX-2 and iNOS expression was inhibited by PEP-1-PON1 and the degree of inhibition increased with increasing concentrations of PEP-1-PON1 (Figs. 3A and 3B). Also, the increased pro-inflammatory cytokine levels induced by LPS were markedly reduced in PEP-1-PON1 protein treated cells. However, cells treated with control PON1 protein did not show reduced cytokine levels compared to those treated with LPS alone (Fig. 3A and Fig. 3B). PEP-1 peptide alone also did not show protective effect against LPS-induced cytokine levels (data not shown). We examined the high (1 µg/ml) LPS-induced inflammation response to clarify the protective effects of PEP-1-PON1 protein. PEP-1-PON1 protein inhibited high LPS-induced inflammatory response and this effect was similar to that of 10 ng/ml LPS (Fig. 3B), suggesting that PEP-1-PON1 protein inhibits LPS-induced inflammatory response in Raw 264.7 cells.

### PEP-1-PON1 Protein Inhibits LPS-induced MAPK and NF-*k*B Activation in Raw 264.7 Cells

**Figure 4:**
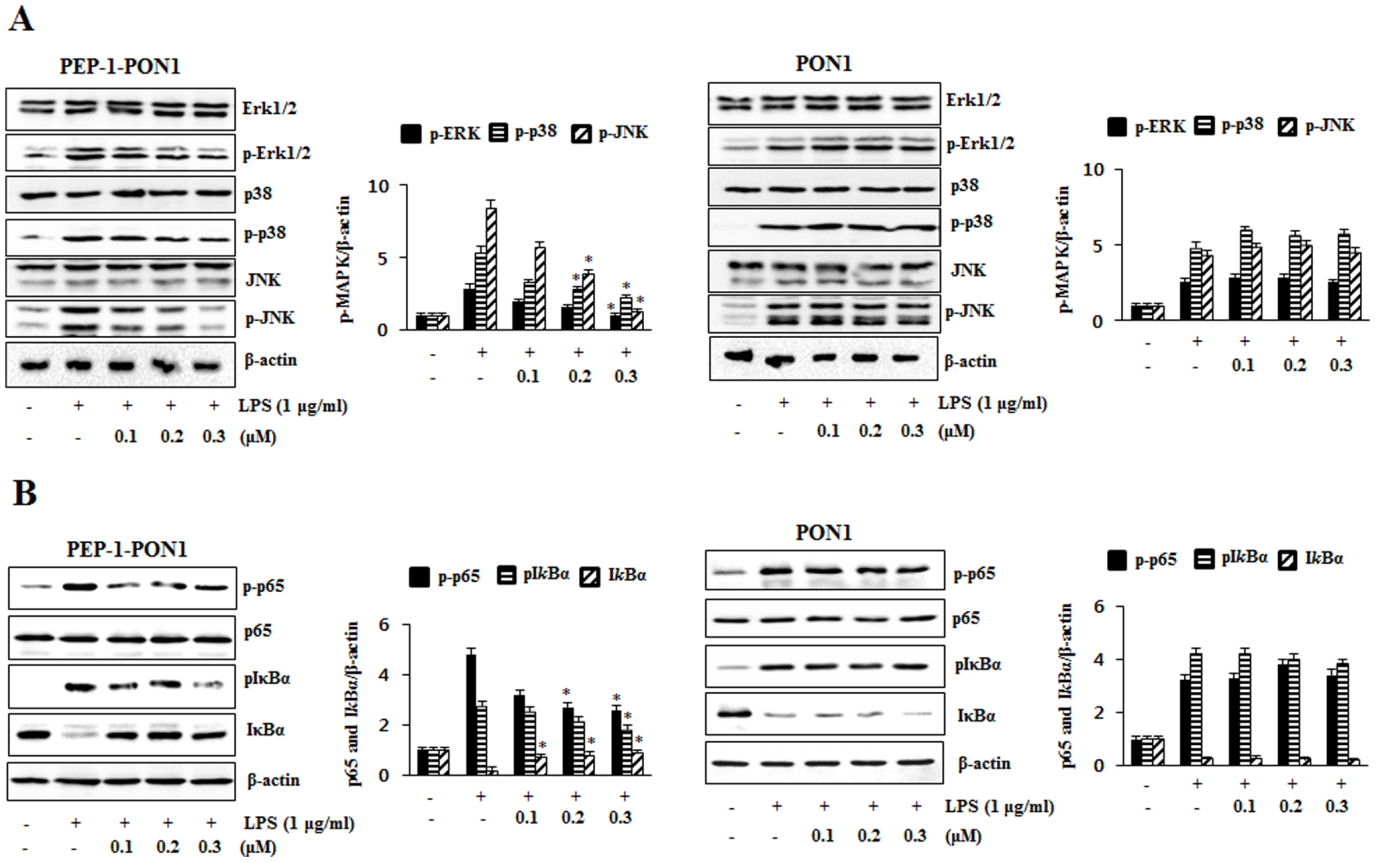
Inhibitory effect of PEP-1-PON1 on LPS-induced MAPK and NF-*k*B activations. Raw 264.7 cells were stimulated with 1 µg/ml LPS for 15 min with or without pretreatment with PEP-1-PON1 protein for 1 h. Cells extract prepared and analyzed for MAPK protein activation by Western blotting and band intensity by densitometer (A). Phosphorylation and the degradation of p65 and I*k*Bα were analyzed by Western blotting and band intensity by densitometer (B). ‘p’ indicates the phosphorylated form of the protein. *P<0.01, compared with LPS treated cells.

LPS induces inflammation in the Raw 264.7 cells by the activation of MAPKs and NF-*k*B [39]. To clearly examine the protective effect of PEP-1-PON1 protein on LPS-induced MAPKs and NF-*k*B activation, the cells were treated with 1 µg/ml LPS for 15 min. As shown in Fig. 4A, ERK1/2, p38, and JNK phosphorylation expression levels markedly increased in the cells treated with LPS alone. However, transduced PEP-1-PON1 protein significantly reduced phosphorylated ERK1/2, p38, and JNK expression levels. Also, transduced PEP-1-PON1 protein markedly inhibited LPS-induced p65 and I*k*Bα phosphorylation levels in the cells (Fig. 4B) whereas LPS-induced MAPKs and NF-*k*B activation were not affected by control PON1 protein. PEP-1 peptide alone showed the similar pattern compared to those treated with LPS alone (data not shown).

### PEP-1-PON1 Protein Inhibits Oxidative Stress-induced Cell Death

**Figure 5:**
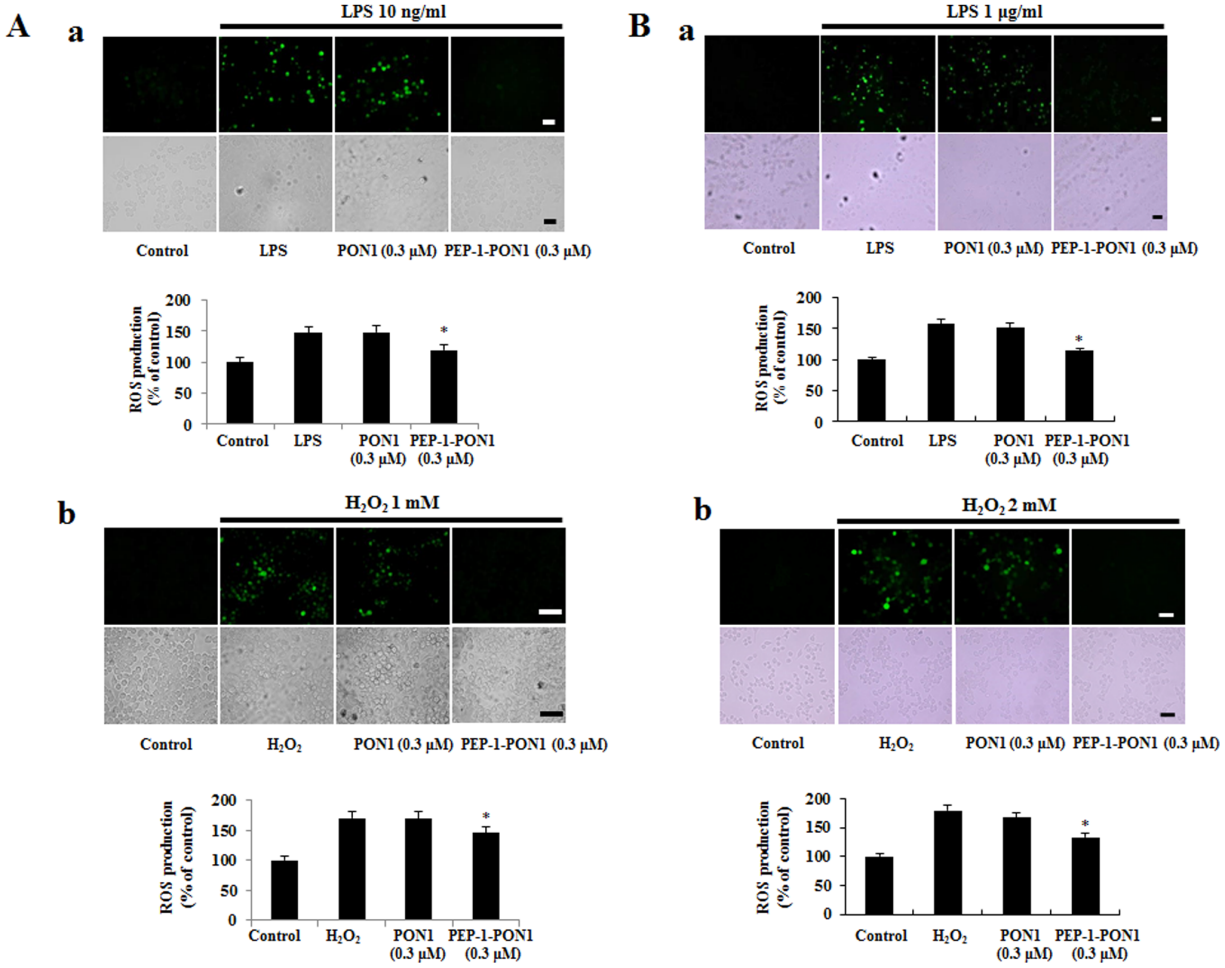
Effect of PEP-1-PON1 on LPS- or H_2_O_2_-induced ROS production. After the cells were treated with PEP-1-PON1 protein for 1 h, cells were stimulated with LPS 10 ng/ml for 50 min and 1 µg/ml for 30 min or H_2_O_2_ 1 mM for 20 min and 2 mM for 10 min. Intracellular ROS levels were measured after staining with DCF-DA and the fluorescent intensity was measured by an ELISA plate reader. Scale bar = 25 µm. *P<0.01, compared with only LPS or H_2_O_2_ treated cells.

LPS induced inflammatory mediators and oxidative stress such as reactive oxygen species (ROS). Excessive ROS leads to cell death [15], [40]. Thus, we examined the LPS-induced ROS production and inhibitory effect of PEP-1-PON1 protein against LPS- or H_2_O_2_-induced ROS in cells using DCF-DA staining. In the cells treated with 10 ng/ml LPS for 50 min, the fluorescence signals were strongly stained by LPS as compared to untreated control cells, whereas transduced PEP-1-PON1 protein reduced the fluorescence signals (Fig. 5A). In addition, in the high LPS (1 µg/ml) treated cells, the fluorescence signals were more strongly stained compared with 10 ng/ml LPS treated cells (Fig. 5B). However, the fluorescence signals showed similar pattern between the 10 ng/ml LPS treated cells and 1 µg/ml LPS treated cells.

Furthermore, we examined ROS production in the cells treated with H_2_O_2_ (1 mM for 20 min or 2 mM for 10 min). As shown in Fig. 5, we observed that the fluorescence signals demonstrated a similar pattern to those of LPS treated cells. Also, control PON1 protein did not show the protective effect in the same experimental conditions. These results show that transduced PEP-1-PON1 protein plays a protective role in LPS- or H_2_O_2_ treated cells by decreasing ROS levels.

**Figure 6:**
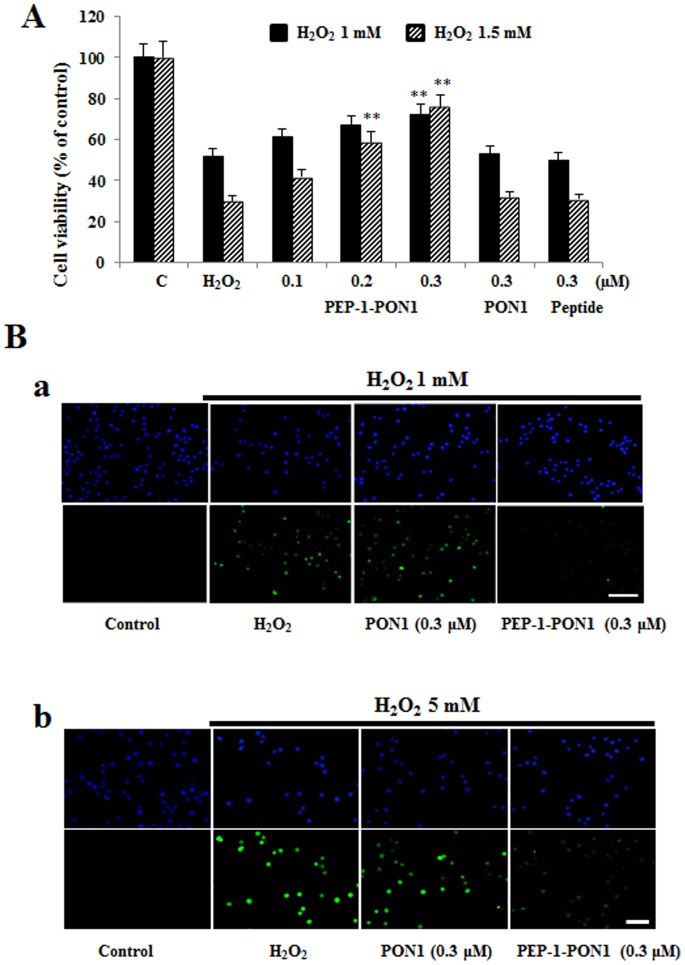
Effect of transduction of PEP-1-PON1 proteins against H_2_O_2_-induced cell viability and DNA fragmentation. H_2_O_2_ (1 mM and 1.5 mM, 16 h) was added to Raw 264.7 cells pretreated with PEP-1-PON1 (0.3 µM) for 1 h. Cell viabilities were estimated by with a colorimetric assay using MTT (A). **P<0.01, compared with H_2_O_2_ treated cells. Cells were treated with PEP-1-PON1 protein (0.3 µM) for 1 h, and then exposed to H_2_O_2_ (1 mM for 15 h and 5 mM for 4 h). DNA fragmentation was detected by TUNEL staining (B). Scale bar = 50 µm.

To determine the protective effects of PEP-1-PON1 protein against cell death, cell viability was measured after exposure to H_2_O_2_ (1 mM or 1.5 mM) for 16 h. As shown in Fig. 6A, cell viability was decreased by H_2_O_2_. Cell viability was significantly increased by transduced PEP-1-PON1 protein in a dose-dependent manner up to 72% and 76%. Next, we examined DNA fragmentation by TUNEL staining. As shown in Fig. 6B, the fluorescence signals in H_2_O_2_ (1 mM for 15 h or 5 mM for 4 h) treated cells was increased compared with the control cells, whereas the fluorescence signals of cells treated with transduced PEP-1-PON1 protein was decreased. However, the fluorescence signals in control PON1 protein treated cells were similar to those of H_2_O_2_ treated control cells. PEP-1 peptide alone did not affect generation of ROS and DNA fragmentation in H_2_O_2_ treated cells (data not shown). Thus, the transduced PEP-1-PON1 protein was biologically active and protects against H_2_O_2_-induced cell death by inhibiting ROS production and DNA fragmentation.

**Figure 7:**
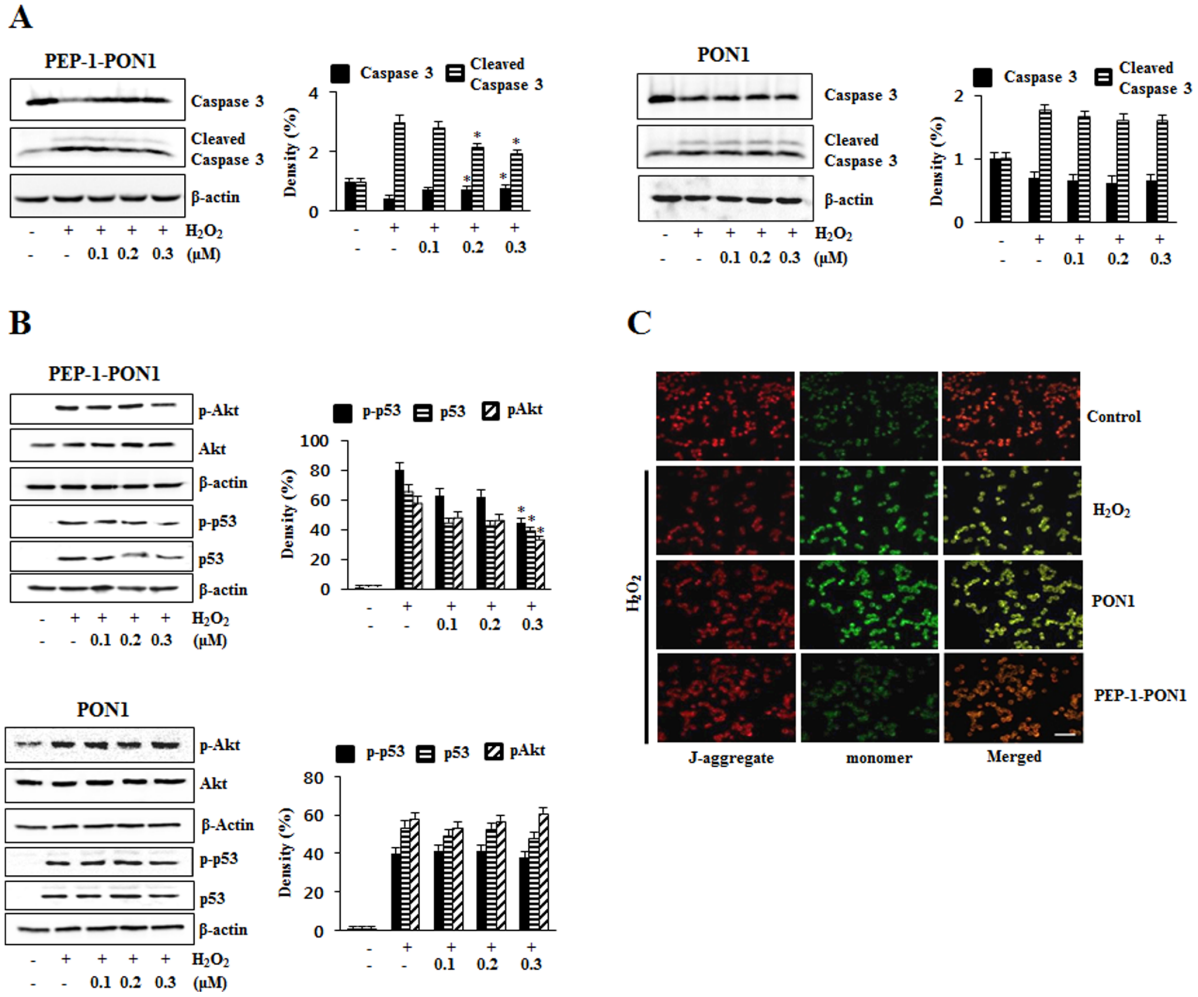
Effect of transduced PEP-1-PON1 protein against H_2_O_2_-induced activation of caspase-3 and mitochondrial membrane potential. Cells were treated with PEP-1-PON1 protein (0.1–0.3 µM) for 1 h, and then exposed to H_2_O_2_. Caspase-3 and cleaved caspase-3 was detected by Western blotting and band intensity by densitometer (A). Akt, p53, phosphorylation Akt and p53 were protein expression levels were measured by Western blotting and band intensity by densitometer (B). *P<0.01, compared with H_2_O_2_ treated cells. ‘p’ indicates the phosphorylated form of the protein. Mitochondrial membrane potential was detected using a mitochondrial membrane potential assay kit (C). Scale bar = 50 µm.

To further confirm the effect of PEP-1-PON1 protein against H_2_O_2_-induced cell death, we examined the activation of caspase-3, Akt, p53 and mitochondrial membrane potential. As shown in Fig. 7A, the activation of caspase-3 was significantly increased by H_2_O_2_. However, PEP-1-PON1 protein markedly reduced the level of cleaved caspase-3. We observed that the levels of phosphorylated Akt and p53 expression were significantly increased in H_2_O_2_-treated cells, while being markedly reduced in the PEP-1-PON1 protein treated cells. However, the levels of phosphorylated Akt and p53 expression were unchanged in control PON1 protein treated cells (Fig. 7B). In addition, PEP-1-PON1 protein treated cells showed an increase in mitochondrial membrane potential compared with H_2_O_2_ treated cells (Fig. 7C). PEP-1 peptide alone did not affect the activation of caspase-3, Akt, p53 and mitochondrial membrane potential in the same experiment conditions (data not shown). These results demonstrate that transduced PEP-1-PON1 protein protected against H_2_O_2_-induced cell death by the inhibiting the apoptotic pathway.

### Effect of PEP-1-PON1 Protein against Inflammation in Animal Model

**Figure 8:**
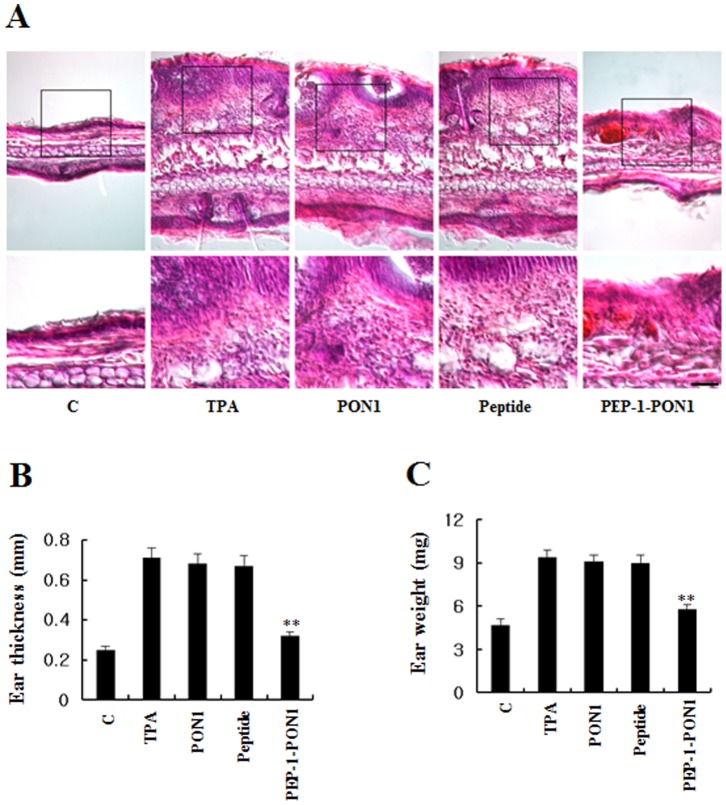
Effect of transduced PEP-1-PON1 protein on TPA-induced ear edema. Ears of mice were treated with TPA (1 µg/ear) and PEP-1-PON1 protein and controls (PON1 protein and PEP-1 peptide) was topically applied to mice ears 1 h after TPA treatment. The inhibition of TPA-induced ear edema was analyzed by hematoxylin and eosin immunostaining (A) and measuring changes in ear thickness (B) as well as weight of 5 mm ear biopsy (C). Scale bar = 50 µm for A (top panel), except for 25 µm in high magnifications in A (bottom panel). **P<0.01, compared with TPA treated mice.

To determine whether PEP-1-PON1 protein protects against skin inflammation, we used a TPA-induce mouse ear edema model [33], [35]. After topical application of TPA and PEP-1-PON1 proteins, we analyzed the ear thickness and weight of 5 mm ear biopsies and their immunohistochemisty. As shown in Fig. 8, PEP-1-PON1 protein significantly inhibited increases in ear thickness and weight of 5 mm ear biopsies induced by TPA. Also, PEP-1-PON1 protein markedly inhibited infiltration of inflammatory cells such as monocytes that is one of the early events in skin inflammation. However, control PON1 protein did not show the same effects.

**Figure 9:**
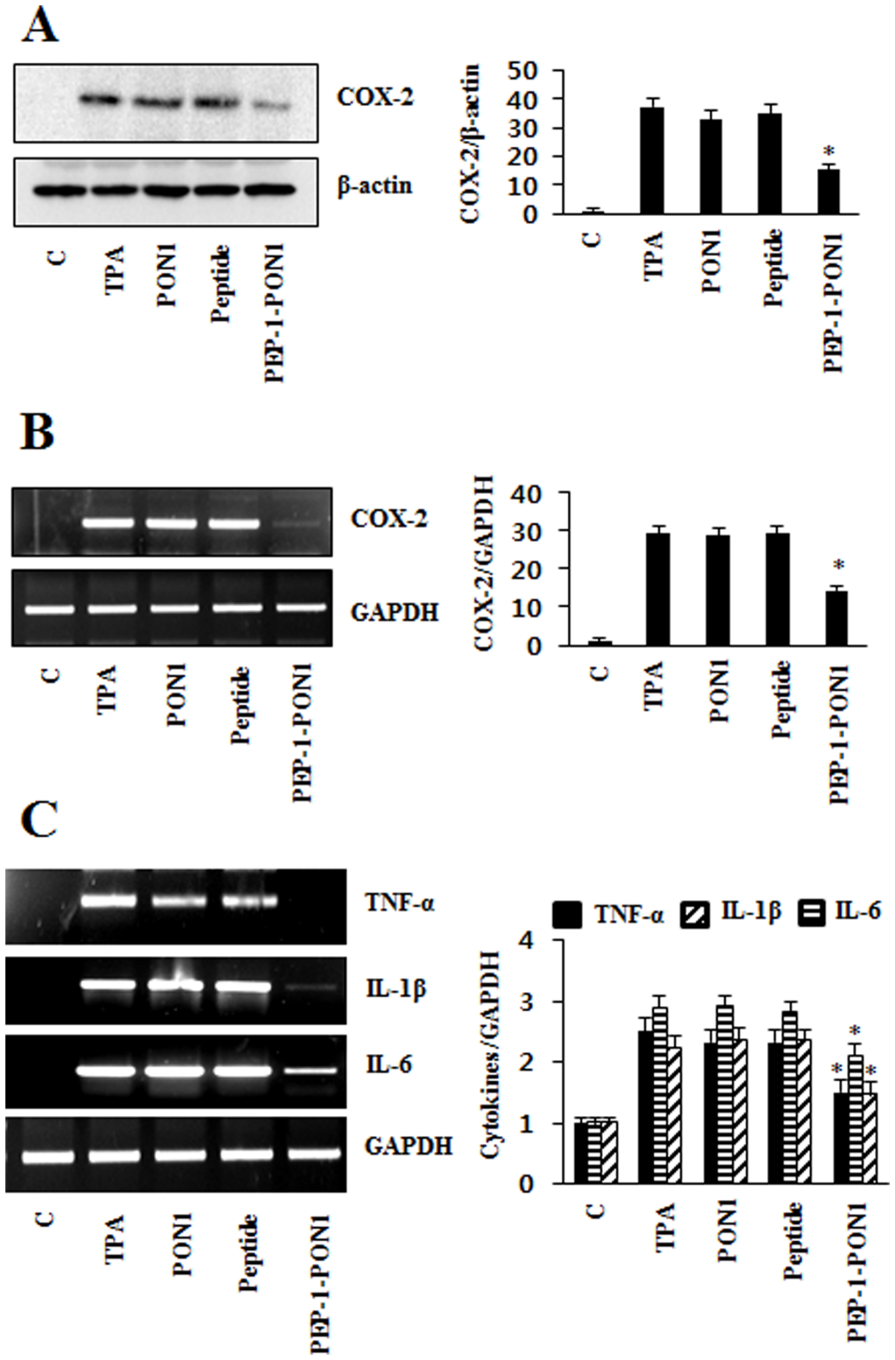
Inhibitory effect of PEP-1-PON1 protein against TPA-induced COX-2 and cytokine expression in edema model. Mice were stimulated with TPA (1 µg/ear) and PEP-1-PON1 protein topically applied to mice ear. Mice ear extracts were prepared and analyzed for COX-2 protein (A) and COX-2 mRNA (B) expression by Western blotting and RT-PCR using specific primers. The band intensity was measured by densitometer. Total RNA was extracted from ear biopsies. TNF-α, IL-1β, IL-6, and GAPDH mRNA were analyzed by RT-PCR using specific primers and band intensity by densitometer (C). *P<0.01, compared with TPA treated mice.

Next, we examined the effect on the expression of COX-2 and cytokine levels in the TPA-induced animal model. TPA significantly increased COX-2 mRNA and protein expression levels. Also, TNF-α, IL-1β, and IL-6 production were increased by TPA. However, PEP-1-PON1 protein markedly reduced expression levels of COX-2 and production of TNF-α, IL-1β, IL-6. While control PON1 protein showed no effect on the COX-2 and cytokines levels (Fig. 9).

**Figure 10:**
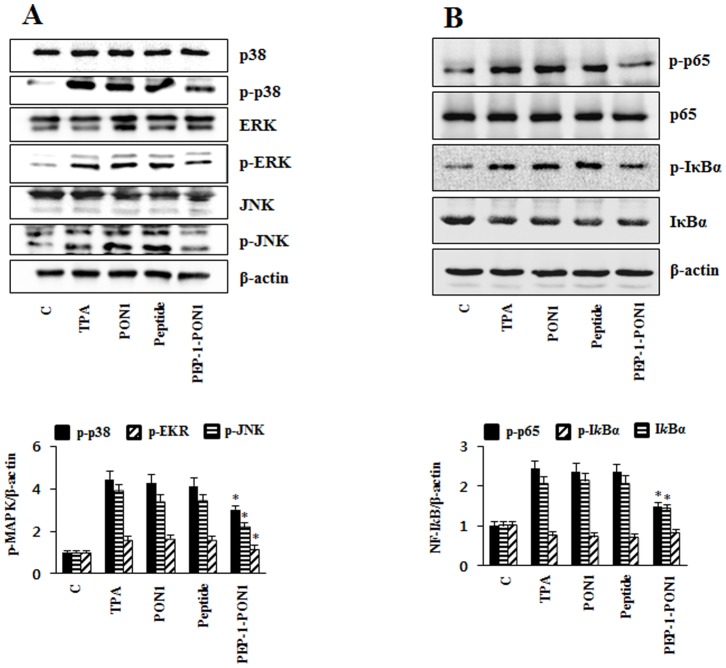
Inhibitory effect of transduced PEP-1-PON1 protein against TPA-induced MAPK and NF-*k*B activation in edema model. Ears of mice were treated with TPA (1 µg/ear) and PEP-1-PON1 protein was topically applied to mice ears 1 h after TPA treatment. Ear biopsies were prepared and analyzed for MAPK protein activation by Western blotting and band intensity by densitometer (A). The degradation and phosphorylation of p65 and I*k*Bα was analyzed by Western blotting and band intensity by densitometer (B). ‘p’ indicates the phosphorylated form of the protein. *P<0.01, compared with TPA treated mice.

We also examined the effects of PEP-1-PON1 protein on the activation of MAPKs and NF-*k*B by Western blotting. PEP-1-PON1 protein inhibited TPA-induced p38, ERK1/2, and JNK phosphorylation as well as p65 and I*k*Bα phosphorylation in the TPA-induced skin inflammation animal model. However, control PON1 protein did not affect MAPK and NF-*k*B activation (Fig. 10). Collectively, these results indicate that transduced PEP-1-PON1 proteins inhibit inflammation through regulation of MAPK and NF-*k*B activation.

## Discussion

Inflammation has been associated with various human diseases including cancer, neurodegenerative diseases, and diabetes [41]–[43]. In addition, inflammatory enzymes (cyclooxygenase-2; COX-2 and inducible nitric oxide synthase; iNOS), cytokines (interleukin-1β; IL-1β and IL-6, tumor necrosis factor-α; TNF-α) and reactive oxygen species (ROS) contribute to cell death and the pathology of various human diseases [18], [44]. Thus, regulation of these factors is important for inflammation and inflammation-related diseases. Although PON1 is considered an antioxidant enzyme and plays a beneficial role in various diseases [1], the functions of PON1 protein in macrophage Raw 264.7 cells and in an inflammation animal model have not been well documented. In this study, we demonstrated that cell permeable PEP-1-PON1 protein inhibits LPS-and TPA-induced inflammation and oxidative stress-induced cell damage *in vitro* and *in vivo* by anti-inflammatory and anti-oxidant effects.

Protein delivery into cells is an important factor in protein therapy. Thus, we generated a cell permeable PEP-1-PON1 protein using protein transduction domains (PTDs). Although the mechanism requires further study, many studies have demonstrated that therapeutic PTD fusion proteins delivered into cells and tissues protect against cell toxicity. Also, PTD fusion proteins have potential in the treatment of various diseases [22]–[27]. In a previous study, we also demonstrated that PEP-1 fusion proteins transduced into cells [28]–[36].

Detoxi-gel is considered as a tool for removing the effects of LPS and the Limulus amebocyte lysate assay is widely used for endotoxin detection [45], [46]. Our study demonstrated that purified PEP-1-PON1 protein was further purified using Detoxi-gel endotoxin removing gel in order to eliminate endotoxin in bacteria (Fig. 1). After purification, endotoxin levels were below the detection limit (<0.1 EU/ml) as tested by the Limulus amebocyte lysate assay (BioWhitaker, Walkersville, MD, USA). In addition, recently reports suggest that the microfiltration is a reliable and highly advantageous tool for the decontamination of samples [45], [46].

Many studies have demonstrated that the overproduction of COX-2, iNOS and pro-inflammatory cytokines induce inflammatory diseases. In addition, the MAPK signal pathway leads to the production of pro-inflammatory cytokines by NF-*k*B activation in macrophage cells [47]–[51]. Thus, we examined the effects of transduced PEP-1-PON1 protein on the LPS-induced expression levels of COX-2, iNOS and pro-inflammatory cytokines in Raw 264.7 cells. In a previous study, we showed that transduced PEP-1-SIRT2 protein protects against LPS-induced inflammatory response or H_2_O_2_-induced oxidative stress in immune cells [33]. Also, other studies have shown that natural products and aldose reductase inhibit LPS- or H_2_O_2_-induced inflammatory response and oxidative stress in immune cells. However, the authors used various incubation times and concentrations of LPS or H_2_O_2_ in these studies [52]–[55]. Thus, we also performed the experiments using various concentrations of LPS or H_2_O_2_ and incubation times prior to do this experiments (data not shown) and finally we determined the optimal concentrations and incubation times of LPS or H_2_O_2_ to observe LPS- or H_2_O_2_-induced inflammatory response and oxidative stress. In this study, we showed that transduced PEP-1-PON1 protein significantly inhibited LPS (10 ng/ml)-induced inflammatory enzymes such as COX-2 and iNOS as well as cytokine (TNF-α, IL-1β, and IL-6) expression levels in Raw 264.7 cells in a dose-dependent manner. In addition, inhibition of COX-2, iNOS and cytokines showed similar patterns in the cells treated with 1 µg/ml LPS (Fig. 3). We also showed that transduced PEP-1-PON1 protein markedly inhibited the LPS-induced activated macrophages intracellular signaling pathway, MAPK pathway and NF-*k*B activation. As shown in Fig. 4, PEP-1-PON1 protein inhibited phosphorylation of MAPK (p38, ERK and JNK) and NF-*k*B (p65 and I*k*Bα) by induce LPS. These results suggest that transduced PEP-1-PON1 protein regulated the NF-*k*B and MAPK pathways by inhibiting LPS-induced inflammatory enzymes and cytokines in the cells.

It is well known that oxidative stress is an important factor in cellular damage, and excessive production of reactive oxygen species (ROS) contributes to cell death and various human diseases related with inflammation [19]. We therefore examined the effects of transduced PEP-1-PON1 protein against oxidative stress-induced cell death. Transduced PEP-1-PON1 protein inhibited LPS- or H_2_O_2_-induced ROS production (Fig. 5). We observed that transduced PEP-1-PON1 protein increased cell viability in a dose-dependent manner compared to H_2_O_2_ treated cells and also protected against DNA fragmentation compared to H_2_O_2_ treated cells (Fig. 6). Recent studies have shown that recombinant PON1 protein dramatically inhibited the pro-inflammatory cytokines in macrophages by suppressing TLR4 activation and NF-*k*B activation. Also, recombinant PON1 protein has a protective effect against apoptosis and ROS production in macrophages, suggesting that further study is needed to understand how PON1 functions on atherosclerosis. PON1 has an anti-inflammatory effect and it may have a possible role as a therapeutic protein [56]. It has also been reported that mitochondria dysfunction and activation of caspase-3 play an important role in cell death [33], [57]. In this study, we demonstrated that transduced PEP-1-PON1 protein protected against cell death by regulating cleaved caspase-3 and mitochondria membrane potential. Also, increased phosphorylations of Akt and p53 protein levels induced by oxidative stress were decreased by exposure to transduced PEP-1-PON1 protein (Fig. 7). Several studies have shown that PON1 protein plays a central role in various diseases by inhibiting oxidative stress. Although further study is needed to understand the exact mechanism, many reports strongly suggest PON1 protein has a potential therapeutic role for various human diseases [1], [11], [57]–[59]. In agreement with other groups, these results indicate that transduced PEP-1-PON1 protein has an anti-oxidant function against oxidative stress-induced cell death.

A 12-O-tetradecanoylphorbol-13-acetate (TPA)-induced ear edema model is well established in the examination of the effect of topical anti-inflammatory treatments [38], [60], [61]. Thus, we examined that the inhibitory effects of PEP-1-PON1 protein against TPA-induced skin inflammation. TPA-treated mice ears were swollen and demonstrated significantly increased thickness and weight of 5 mm ear biopsies whereas the topical application of PEP-1-PON1 protein inhibited the TPA-induced increase in thickness and weights (Fig. 8). Pro-inflammatory cytokines (TNF-α, IL-1β, and IL-6) are induced by TPA and play important roles including the regulation of MAPK and NF-*k*B [38], [62], [63]. In the TPA-induced inflammation animal model, we demonstrated that topical application of PEP-1-PON1 protein significantly inhibited pro-inflammatory cytokines and COX-2 expression levels (Fig. 9). In addition, topical application of PEP-1-PON1 protein inhibited TPA-induced phosphorylation/degradation of NF-*k*B and I*k*Bα as well as phosphorylation of MAPKs (p38, ERK and JNK) in mice ears (Fig. 10). These results indicate that PEP-1-PON1 protein has an anti-inflammatory effect in LPS-induced macrophage cells and TPA-induced skin tissues by regulating the signaling pathways through the activation of NF-*k*B and MAPK.

In the present study, we revealed that transduced PEP-1-PON1 protein has anti-inflammatory and anti-oxidant effects *in vitro* and *in vivo* by regulating inflammatory response and oxidative stress. Therefore, we suggest that PEP-1-PON1 protein may be a potential therapeutic agent for inflammation and ROS-related diseases.
